# Unveiling the diversification and dispersal of the *Lewinskya firma* complex (Orthotrichaceae, Bryophyta) across Africa and India

**DOI:** 10.3389/fpls.2024.1451005

**Published:** 2024-09-27

**Authors:** Francisco Lara, Raúl Díaz San Román, Mario Fernández-Mazuecos, Juan Antonio Calleja, Maren Flagmeier, Vicente Mazimpaka, Ricardo Garilleti, Isabel Draper

**Affiliations:** ^1^ Departamento de Biología, Facultad de Ciencias, Universidad Autónoma de Madrid, Madrid, Spain; ^2^ Centro de Investigación en Biodiversidad y Cambio Global, Universidad Autónoma de Madrid, Madrid, Spain; ^3^ Departamento de Botánica y Geología, Universidad de Valencia, Valencia, Spain

**Keywords:** biogeography, intercontinental disjunction, mosses, *Orthotrichum firmum*, taxonomy, speciation

## Abstract

Intercontinental disjunctions are one of the most attractive and interesting biogeographical patterns. Bryophytes often exhibit such distributions, which is partly explained by their great ability to disperse over long distances. However, such intercontinental ranges are sometimes a distorted reality caused by the existence of unnoticed species. This study investigates whether the disjunction between East Africa and southern India of the moss *Lewinskya firma* reflects the genuine distribution of a single species or implies pseudo-cryptic species (whose morphological differentiation is subtle and have therefore been masked). An integrative taxonomic approach combining morphological and molecular methods (genotyping by sequencing, GBS) was used, based on a representation of samples specifically collected from all the major mountainous regions where this moss occurs. Two species, *L. firma* s. str. and *L. afroindica* sp. nov. are involved, whose ranges completely overlap in East Africa, although genetic distance and morphological differences in leaf apex shape, vaginula hairs shape and papillosity, spore ornamentation and peristome constitution and ornamentation allow distinguishing both. In addition, the range of *L. afroindica* extends into both southern Africa and southern India. The phylogenetic reconstruction obtained shows a certain degree of differentiation of the Indian populations, although they are yet morphologically indistinguishable from African populations. The results thus highlight both the existence of overlooked species and the complexity of bryophyte biogeography.

## 1 Introduction

Bryophytes, including mosses, liverworts and hornworts, are a major lineage of land plants whose diversity and distribution patterns are not yet fully understood (Goffinet and Shaw, 2009). Most bryophyte species have broad distribution ranges, often spanning areas of more than one continent (Schofield and Crum, 1972; Patiño and Vanderpoorten, 2018). Some remarkable intercontinental disjunctions displayed by species of bryophytes coincide with those observed in genera or families of other terrestrial plants, which has traditionally led to the consideration of continental drift as a possible common cause (Schofield, 1992; Heinrichs et al., 2009). Nevertheless, in the last decades, it has become increasingly evident that most disjunctions among bryophytes are due to long-distance dispersal processes—while also recognizing its importance in seed plants (e.g., Pokorny et al., 2015; Lopes et al., 2024)—as molecular dating does not align with ancient vicariance processes (Muñoz et al., 2004; Heinrichs et al., 2009; Patiño and Vanderpoorten, 2018; Flagmeier et al., 2020, Flagmeier et al., 2021). Furthermore, the wide distribution patterns of bryophytes have been related to their capacity to produce large numbers of microscopic spores capable of being transported over long distances, mainly by wind (Muñoz et al., 2004; Vigalondo et al., 2016; Estébanez et al., 2018).

However, certain species previously considered widespread have been found to reflect taxonomic shortcomings, as they are now understood to represent complexes of two or more pseudo-cryptic species (in the sense that morphological differentiation was overlooked). The resulting species are not necessarily closely related to each other and often exhibit narrow distribution ranges (Heinrichs et al., 2010; Stech et al., 2013; Heinrichs et al., 2015; Lang et al., 2015; Carter et al., 2017; Vigalondo et al., 2019a; Hedenäs, 2020). Addressing and resolving such complexes of species represents a challenge (Fišer et al., 2018), especially when studying small-sized organisms with reduced morphological complexity, as is the case of many bryophytes (Bickford et al., 2007; Fontaneto, 2011). Integrative taxonomy has proven highly effective for solving taxonomic puzzles of cryptic or pseudo-cryptic species complexes within bryophytes (Medina et al., 2012; Medina et al., 2013; Heinrichs et al., 2015; Caparrós et al., 2016; Sim-Sim et al., 2017; Vigalondo et al., 2019a; Hanusch et al., 2020).

Among bryophytes, one of the most speciose families is Orthotrichaceae Arn., with ca. 800 accepted species (Brinda and Atwood, 2023), being the second most diverse group of mosses. This group also displays a highly diverse range of species distribution patterns. Species known from a single continent are predominant, and those limited to a specific biogeographic region are not uncommon (Lewinsky, 1993). Despite the relative rarity of extremely narrow distributions among bryophytes (Medina et al., 2011), several examples of local endemics are also known (e.g., Patiño et al., 2013). Conversely, many species of Orthotrichaceae exhibit remarkably wide ranges, including intercontinental disjunct distributions. Several taxonomic integrative studies have confirmed the existence of species whose distribution genuinely involves more than one continent (Caparrós et al., 2016; Vigalondo et al., 2016; Vigalondo et al., 2019b; Flagmeier et al., 2021). However, there are other cases where complexes of pseudo-cryptic species, restricted to a single continent or archipelago, have been unveiled (Medina et al., 2012, 2013; Vigalondo et al., 2019a; Lara et al., 2020). Notably, every case examined to date corresponds to species or species complexes with disjunct areas in the Holarctic realm, generally involving North America on one end and Europe and the Mediterranean on the other. However, there are examples of disjunct distributions affecting other areas of the world that equally deserve a deeper investigation (Vitt, 1980; Vitt and Ramsay, 1985; Lewinsky, 1993; Garilleti et al., 2015).

A suggestive tropical disjunction affecting East Africa and southern India is shown by the orthotrichaceous moss *Lewinskya firma* (Venturi) F.Lara, Garilleti & Goffinet. In Africa, it is a widespread epiphytic species living in forests and shrublands of the Afromontane and Afroalpine belts between 1500 and 4500 m of elevation (Lewinsky and van Rooy, 1990). Its distribution, as known today (Lewinsky, 1978; O’Shea, 2006), encompasses much of East sub-Saharan Africa, albeit in a discontinuous manner. The species is confined to ‘sky islands’ within the Afromontane region (White, 1978; Carbutt and Edwards, 2015), spanning ca. 48° of latitude, from the Eritrean and Ethiopian Highlands, throughout the Great Rift Mountains to the eastern portion of the South African Great Escarpment. This area represents a fragmented ecological system, also referred to as the Afromontane and Afroalpine Archipelagos (White, 1981; White, 1983), where populations are isolated from each other by tropical lowlands (Chala et al., 2017).

On the other hand, a population of *Lewinskya firma* from India was discovered in Ootacamund (Udhagamandalam), a locality in the Nilgiri Hills in northwestern Tamil Nadu State, near the southernmost tip of the Indian subcontinent (Lewinsky, 1980). The area corresponds to the Southern Western Ghats and lies in one of the few zones that exceed 2200 m of elevation in the so-called Great Escarpment of India (Migon, 2010). It is a tropical altimontane environment, although the specified location is a highly populated area, surrounded by a hilly landscape with grasslands and remnants of the tropical montane evergreen forest known as shola-grassland ecosystem mosaic (Mohandass and Davidar, 2009; Bunyan et al., 2012). In India, as in the case of East Africa, *L. firma* grows in high mountains that constitute ecological islands rich in epiphytic mosses that stands out from a tropical lowland environment (Verma et al., 2011).

*Lewinskya firma* is a highly distinctive species even in the field, as no other tropical African or southern Indian species has exserted and smooth capsules with a peristome of sixteen exostome teeth and sixteen wide endostome segments (Lewinsky, 1978, 1980; Magill and van Rooy, 1998). Lewinsky (1978) considered that the variability found in some other morphological features of the species lacked taxonomic relevance; this notion was strengthened by the discovery that specimens from India and Africa exhibited morphological ranges that were entirely comparable (Lewinsky, 1980). We have had the opportunity to collect epiphytic mosses in the major mountainous regions of Africa and India where *L. firma* inhabits and have observed notable variation in certain features among different populations. The most striking variation is the differing prominence of sporophytes on the gametophores or green plants. While in some specimens the capsules are widely exserted, developed on setae of considerable length that surpass the perichaetial leaves, in others the capsules barely extend beyond these perichaetial leaves as they develop on shorter setae.

The peculiar distribution of *Lewinskya firma* and its morphological variability affecting traits visible to the naked eye may suggest that the current concept of *L. firma* could include more than one species, as similarly found for other disjunct orthotrichaceous taxa. To verify this, we carried out an integrative taxonomic study that combined morphological and molecular approaches, including phylogenomic analyses based on genotyping-by-sequencing (GBS). The specific objectives of the study are: 1) to clarify whether the apparent disjunct distribution of *L. firma* between Africa and India is real or, on the contrary, results from the existence of cryptic or pseudo-cryptic species; 2) if there is more than one taxonomic entity, to determine the morphological limits and phylogenetic relationships of the taxa involved; and 3) to understand the distribution patterns of the resulting taxa and check whether they are related to geographic gradients and the discontinuous distribution of available habitats, since these factors could have influenced the speciation of this group of mosses.

## 2 Materials and methods

### 2.1 Morpho-chorological hypothesis and sampling of the complex

**Figure 1:**
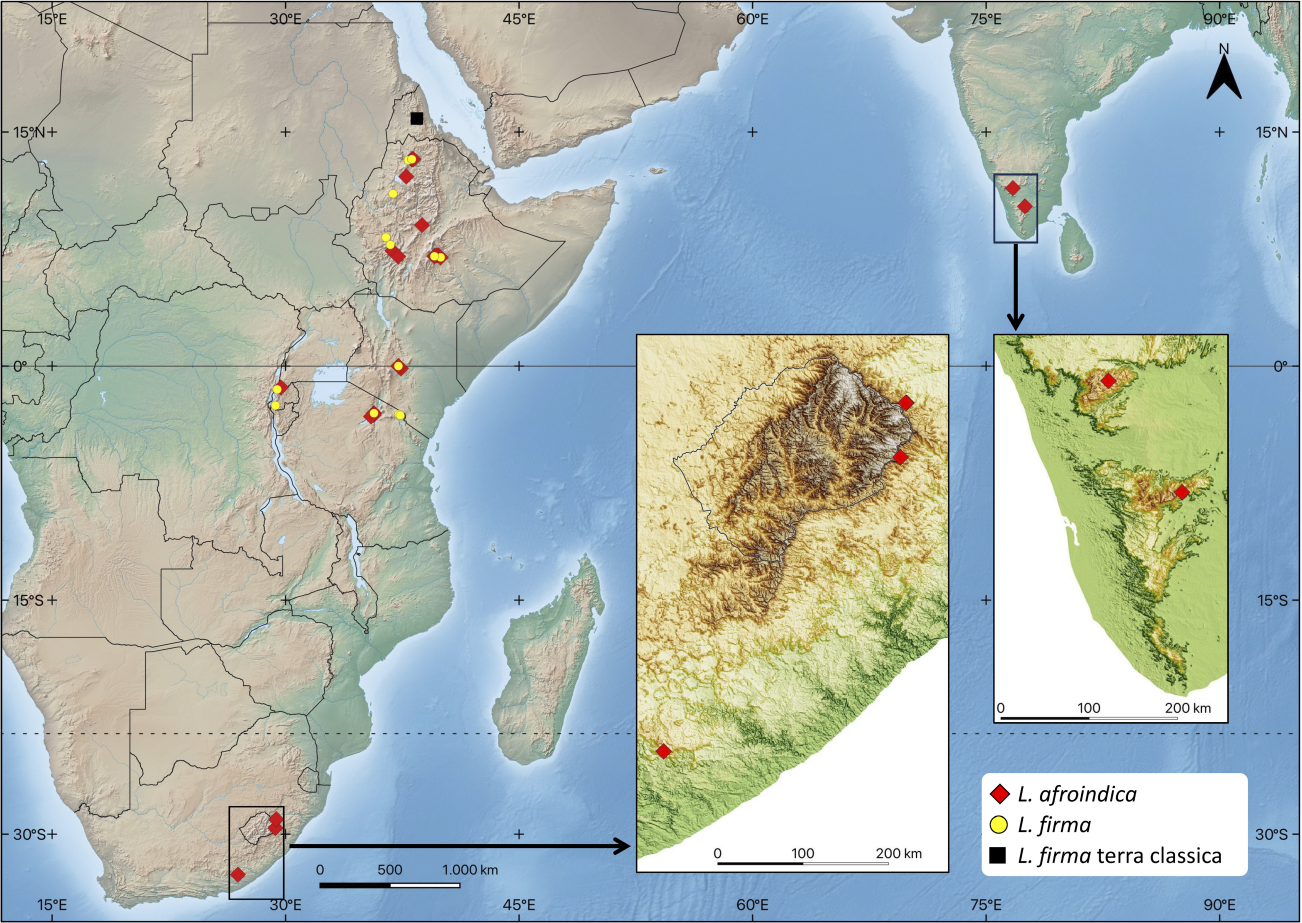
*Lewinskya firma* complex distribution range. The distribution of *Lewinskya firma* and *Lewinskya afroindica* is based on the studied specimens cited in the text. (Map created from base map downloaded from https://www.naturalearthdata.com, and regional DEM downloaded from https://gdemdl.aster.jspacesystems.or.jp).

We studied 107 specimens that encompass the current morphological concept of *Lewinskya firma*, and that cover most of its distribution range (**Figure 1**). These include 59 samples from Ethiopia, 8 from Kenya, 12 from Tanzania, 9 from Rwanda, 9 from South Africa and 10 from India. To test whether the observed morphological variability and/or disjunct populations could represent separate taxa, the samples were initially divided in four morpho-chorotypes according to i) their geographical origin; and ii) a few variable conspicuous morphological features. The four groups defined, hereafter called morphotypes, were: morphotype 1, from Ethiopia and Great Rift Mountains, with leaf apex acute to mucronate and capsules shortly exserted; morphotype 2, from Ethiopia and Great Rift Mountains, with leaf apex channeled-cuspidate and capsule shortly exserted; morphotype 3, from Southern India, with leaves stiff when dry, denticulate at distal end, leaf apex channeled-cuspidate and capsule long exserted; and morphotype 4, from Great Rift Mountains and South Africa, with leaves undulate when dry, not denticulate at distal end, leaf apex channelled-cuspidate and capsule long exserted.

Nearly all the analyzed samples were collected by members of our research team in different campaigns from 2012 to 2023. All vouchers are deposited in the herbarium of the Autonomous University of Madrid (MAUAM) (see **Annex 1** in **Supplementary Material**).

### 2.2 Morphological studies

A set of over 100 morphological characters, both qualitative and quantitative, was studied, selected according to those previously validly used in Orthotrichaceae (Lara et al., 2009; Lara et al., 2014; Medina et al., 2012, 2013; Vigalondo et al., 2016). Qualitative traits of the gametophyte include plant habit and several leaf characters such as shape, margin curvature, apices shape and cell papillosity; calyptra and vaginula hairiness were also evaluated. Sporophyte characters are usually of great diagnostic value for the genus (Vitt, 1973; Lewinsky, 1993). Therefore, we paid special attention to the study of the capsules shape, exothecial band differentiation, stomata position, structure and ornamentation of the peristome, and spore ornamentation. For quantitative characters, we focused on evaluating the size of leaves and their parts, including leaf cells at different locations, the length of the calyptra, seta, capsules, peristome teeth and spore diameter. The morphological characters evaluated are listed at https://www.orthotree.net/orthotrichaceae, and explanations on the used characters can be found in Lara et al. (2009, 2014) and Vigalondo et al. (2019a).

The morphological analysis of the samples was based on light microscope examination. However, selected samples were subsequently examined under scanning electron microscopy (SEM) to confirm peristome and spore details. For observation under SEM, completely dried samples were mounted on aluminum studs and then sputter coated with gold/palladium with a thickness of about 200 Å. Micrographs were obtained using a SCIOS 2 FIB-SEM Field Emission SEM at an accelerating voltage of 3.00 kV.

### 2.3 DNA extraction

A total of 43 specimens representing the four morphotypes were selected for molecular analyses, and sufficient quantity and quality of DNA for GBS library preparation was obtained for a sub-set of 19 samples (**Annex 2** in **Supplementary Material**). Twelve additional samples of 11 *Lewinskya* species from Africa and the Mediterranean area, and based on previous results (Draper et al., 2021), were selected to complete the phylogenetic frame (**Annex 2** in **Supplementary Material**). In Orthotrichaceae, several species morphologically similar frequently coexist in the same cushion. To ensure that only the desired specimen was sampled, DNA was extracted from the apical end of a single gametophore, whenever possible. Only in those cases where the amount of plant material would not be sufficient to obtain optimal DNA quantities for GBS, more than one shoot were selected, as long as they seemed to have originated from the same protonema. In all cases, remains of the gametophyte and the sporophyte were preserved on a permanent microscope slide, mounted with Kaiser’s glycerol gelatine, to enable morphological review if necessary.

DNA was extracted using a modified cetyltrimethylammonium bromide (CTAB) protocol (Doyle and Doyle, 1987) described in Breinholt et al. (2021). The plant material was ground by hand using a mortar and sterilized sand. We performed two rounds of chloroform washes, and 2 μl of 10 mg/ml RNase A (Qiagen, Hilden, Germany) were added to each sample between each chloroform wash to remove RNA contamination. DNA was precipitated in isopropanol overnight in the freezer, or longer if herbarium material was old. Finally, a 70% ethanol wash was performed and DNA eluted in buffer. We quantified the DNA concentrations using a Qubit 4 fluorometer (Invitrogen, CA, USA) with 1X dsDNA high-sensitivity assay kit, following the manufacturer’s protocol.

### 2.4 Genotyping-by-sequencing library preparation and data assembly

Reduced representation libraries were prepared using the *Pst*I-HF restriction enzyme (NEB, MA, USA) following the protocol published by Fernández-Mazuecos et al. (2018) (based on Elshire et al., 2011; Escudero et al., 2014; Grabowski et al., 2014). To allow for multiplex sequencing, each DNA sample was tagged with a unique index identifier sequence (“barcode”), each differing in at least three base substitutions, designed using the Deena Bioinformatics GBS Barcode Generator [available in December 2018 at http://www.deenabio.com/services/gbs-adapters (**Annex 2** in **Supplementary Material**)]. The barcode was integrated in an adapter oligonucleotide (barcode adapter), which together with a common adapter (both specific to the restriction enzyme used) flank the DNA fragments generated by the enzymatic fragmentation. To achieve this, for each sample the corresponding volume for 500 ng of DNA (when possible) was combined with 6 μl of a sample-specific working adapter stock (including a barcode adapter and the common adapter, both at 0.1 μM) in a 96-well PCR plate and dried. The samples were then digested with four units of the restriction enzyme *Pst*I-HF at 37°C overnight, in a total volume of 20 μl following the manufacturer’s protocol. Subsequently, adapters were ligated by adding four units of T4 Ligase (NEB, MA, USA) and the corresponding ligase buffer in a reaction volume of 50 μl, incubating at room temperature for 4 h, followed by heating to 65°C for 10 min to inactivate the ligase and therefore prevent misligation after pooling. We pooled 50 ng of DNA of each sample and purified at a ratio of 1:1 Agencourt AMPure XP (Beckman Coulter; Brea, CA, USA). DNA concentration was quantified and 35 ng of DNA used in a 50 μl PCR reaction to perform amplification of restriction fragments with ligated adapters, with primers containing the sequencing binding sites for the Illumina NGS platform, and 2X Taq Master Mix (NEB, MA, USA). The PCR cycles varied between 17 and 21 to find the optimal library profile and concentration. Finally, 1 μl of each PCR product was purified with 0.8:1 volume ratio of AMPure XP beads and run on a 2100 Bioanalyzer (Agilent Technologies; Santa Clara, CA, USA) to evaluate DNA fragment size distribution and concentration. We selected and submitted the library generated with 17 PCR cycles to Macrogen (Seoul, South Korea) for Illumina HiSeq X paired-end (150 bp x 2) sequencing.

The obtained loci were assembled using the ipyrad 0.9.88 pipeline (Eaton and Overcast, 2020). This pipeline was run on the cluster of the Center for Scientific Computing of the Autonomous University of Madrid (CCC-UAM, Madrid, Spain). We conducted a *de novo* assembly due to the lack of a reference genome for the group. The parameters for ipyrad steps used in our study were based on Fernández-Mazuecos et al. (2020), modifying the maximum number of alleles per site to 1 given the haploidy of moss gametophytes. We tested different values for two assembling parameters: the similarity threshold employed for within-sample and across-sample sequence clustering (c), and the minimum sample coverage (m). Thus, we generated six assemblies of GBS loci combining three values of clustering threshold (0.85, 0.90 and 0.95) and two values of the minimum sample coverage (4 and 10). We excluded the individuals with low locus recovery (< 230 loci) in preliminary assemblies and those with uncertain phylogenetic placement in preliminary phylogenetic analyses.

### 2.5 Phylogenomic analyses

Phylogenetic relationships were inferred using Maximum Likelihood (ML) for the six assemblies designed with the parameters explained in 2.4. The analyses were implemented in RAxML-HPC BlackBox 8.2.12 (Stamatakis, 2014) using the CIPRES Science Gateway (Miller et al., 2010) under the default nucleotide substitution model in RAxML (GTR+G), which is commonly used in GBS analyses (Fernández-Mazuecos et al., 2018, 2020; Alonso et al., 2022; Gallego‐Narbón et al., 2023). The number of bootstrap replicates was automatically evaluated by RAxML (Pattengale et al., 2010). The resulting trees were visualized and edited in FigTree 1.4.4 (Rambaut, 2018). Trees were rooted with *L. incurvomarginata* as outgroup following previous phylogenetic evidence (unpublished results). A splits network based on the same matrix was computed in SplitsTree4 (Huson and Bryant, 2006) using the NeighborNet method (Bryant and Moulton, 2004).

## 3 Results

### 3.1 Morphological studies

The morphological features of the studied specimens allow distinguishing two groups that we hereafter name A and B. Group A includes all the specimens initially assigned to morphotype 1, whereas group B includes those corresponding to the other three initial morphotypes (morphotypes 2, 3, and 4). In other words, only morphotype 1 can be clearly separated from the rest by morphological characters. Exclusive characteristics to distinguish both groups appear consistently across traits of the gametophyte and the sporophyte. This allows safe segregation of specimens even in samples from the same locality.

Main morphological features of the two groups are summarized in **Table 1**. Considering only especially helpful characters, specimens corresponding to group A (morphotype 1) present leaves with apex acute to short acuminate, vaginula naked or with scarce slightly papillose hairs, and rugulose, almost smooth spores; conversely, specimens belonging to group B (morphotypes 2, 3 and 4) present leaves with apex acuminate to cuspidate, frequently channeled and occasionally ± dentate, vaginula with numerous papillose or dentate hairs, and papillose spores. Other morphological characters of both groups are indicated in the detailed descriptions included in the Taxonomy section.

**Table 1:** Morphological groups within the *Lewinskya firma* complex, with their main distinctive morphological features.

	Group A	Group B
	Morphotype 1	Morphotypes 2, 3 and 4
**Leaves size (mm)**	2.3–4.2 × 0.6–1.1	2.2–5.0 × 0.7–1.4
**Leaf apex**	Acute to short acuminate or mucronate	Acuminate or cuspidate-channeled, occ. Dentate
**Upper and median leaf cells size (μm)**	8–20 × 7–13	8–28 × 8–25
**Vaginula**	Naked or with isolated hairs, rarely hairy	Hairy, with ± numerous hairs, rarely with isolate hairs
**Vaginula hairs**	Smooth to slightly papillose	Strongly papillose to dentate
**Calyptra length (mm)**	2.5–3.0	2.2–3.6
**Calyptra hairiness**	Openly hairy, occ. densely hairy	Densely hairy, rarely openly hairy
**Calyptra hairs**	Short, thin, moderately papillose	Short, stout, strongly papillose to dentate
**Seta length (mm)**	1.1–3.4	(0.8–)1.1–4.0(–4.7)
**Exostome teeth**	16, independent from the beginning	16, originally paired and quickly splitting, some pairs often remain partially united
**Exostome teeth ornamentation**	Densely covered by tall, thick, simple papillae	Densely covered by granulose papillae
**Exostome teeth position when dry**	Reflexed, leaning against the exothecium, occ. revolute	Revolute, touching the exothecium by the tip
**Endostome segments (as seen under LM)**	Apparently arisen some distance from capsule mouth (above the 3rd-4th basal cells of exostome tooth)	Apparently arisen near the capsule mouth (at most, above the 2nd basal cell of the exostome tooth)
**Spore diameter (μm)**	(22–)26–33	(18–)21–28(–35)
**Spore wall ornamentation**	Smooth or nearly so	Strongly papillose

The specimens belonging to group B were initially divided in three morphotypes (2, 3, and 4) based on the degree of capsule exsertion and on minor variations in the leaf shape. Initially, we considered that these characteristics varied among different geographic areas. However, the morphological study does not support this division since none of the evaluated characters can be ascribed to a unique morphotype or geographic area. Moreover, no morphological trend of consistent association of features offers a differentiating diagnosis to clearly separate the specimens.

Seta length was measured as an approximation to evaluate capsula exsertion from perichaetial leaves in the four morphotypes (**Table 1**). Corresponding values within group B are 0.8–2.5 mm (morphotype 2), (1.0–)1.5–3.8(–4.7) mm (morphotype 3), and 2.6–4.0 mm (morphotype 4). Given the overlapping variability among morphotypes in group B, we analyzed if the differences in seta length could result from a geographical pattern. We have found high variability in this feature in all the considered areas. Nevertheless, specimens showing setae lengths closer to one or another extreme of the general interval dominate in some of the geographic areas, according to our initial morphological hypothesis. Thus, specimens collected in the mountains around the Great Rift display significant variability in this characteristic. Both slightly and long exserted capsules coexist without a clear predominance or any of either type. In contrast, specimens from the northern end of Ethiopia exhibit particularly short setae and slightly exserted capsules, while those from South Africa and the southern tip of India predominantly feature long setae and distinctly exserted capsules.

### 3.2 Phylogenomic inference

The generated library provided 737,950,396 total reads, with a total number of 111,430,509,796 bp. Bases with a quality score of Q20 represented 96.7%. The comparison of the phylogenetic reconstructions obtained from the analyses of the six different datasets (see 2.4) indicated that the best resolved topology and highest node support values were obtained with a clustering threshold value of 0.95 and a minimum sample coverage of 4, with a final dataset of 32 samples. In this dataset, 7,086 filtered loci were retained, with a total number of 963,941 bp, including 33,778 variable sites, of which 10,515 were parsimony informative, and 77.53% of missing sites. Information regarding the number of loci recovered and missing data percentage per sample can be found in **Annex 2** in **Supplementary Material**. Sequences are available at the Sequence Read Archive (NCBI), BioProject PRJNA1134949.

**Figure 2:**
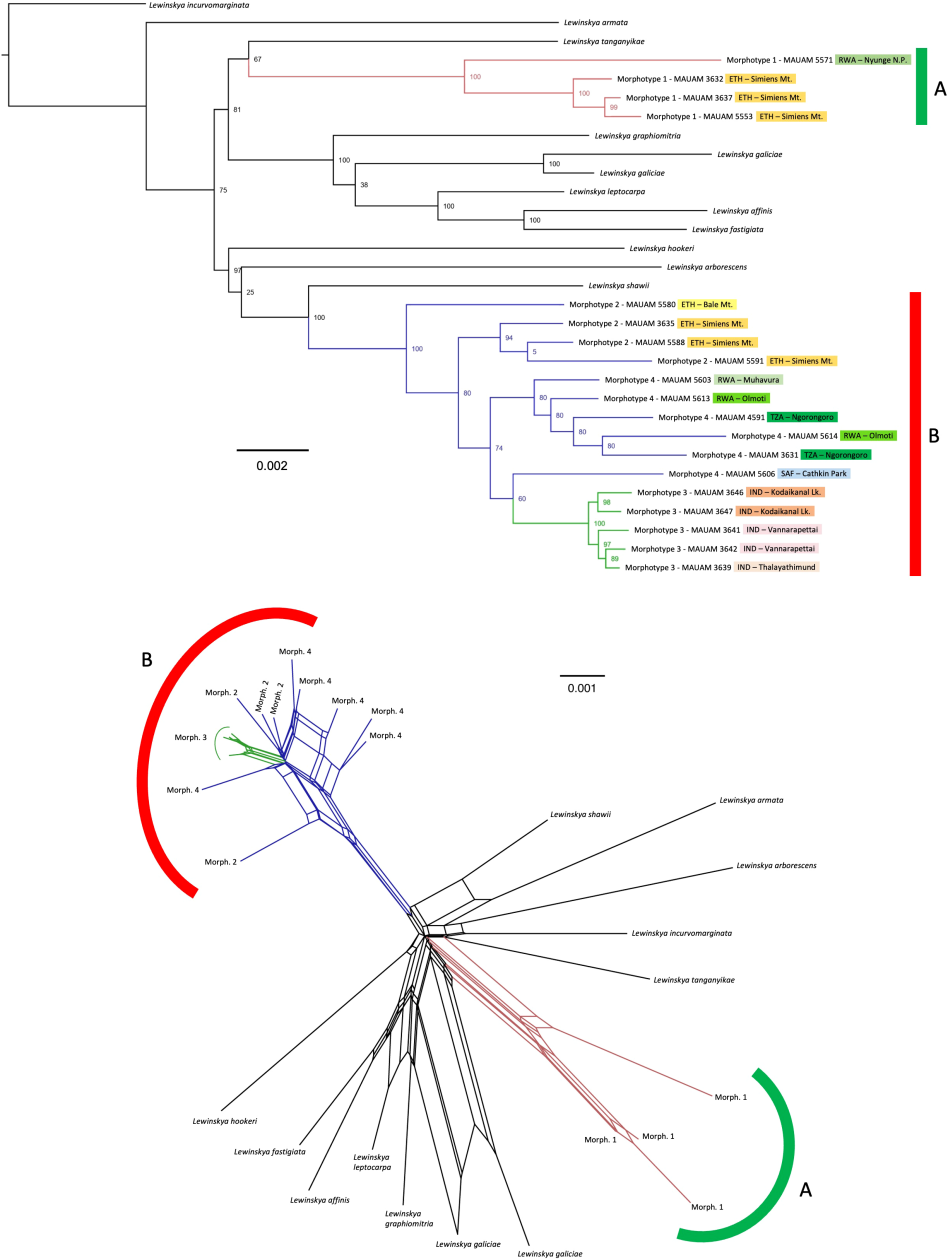
*Lewinskya firma* complex phylogenetic reconstruction, TOP: phylogenetic tree inferred by Maximum Likelihood, based on concatenated DNA sequences of 7,086 loci obtained by genotyping by sequencing (c0.95m4 dataset), with clade bootstrap support indicated beside the nodes. Branch colors indicate the morphotypes (1, red; 2 and 4, blue; and 3, green). ETH, Ethiopia; IND, India; RWA, Rwanda; TZA, Tanzania; SAF, South Africa. BOTTOM: Splits network showing potential phylogenetic conflicts among the samples, with the same branch colors to indicate the morphotypes.

The phylogenetic reconstruction selected is shown in **Figure 2**. *Lewinskya firma* as traditionally understood is recovered as polyphyletic, since it is divided in two clearly separate fully supported clades (BS=100), which correspond to the morphologically defined groups A and B. On the one hand, the clade corresponding to group A (morphotype 1) is apparently sister to *Lewinskya tanganyikae* (P. de la Varde) F.Lara, Garilleti & Goffinet, although this relationship is poorly supported (BS=67). Other closely related species, with stronger support (BS=81), are *L. graphiomitria* (Müll.Hal. ex Beckett) F.Lara, Garilleti & Goffinet, *L. galiciae* (F.Lara, Garilleti & Mazimpaka) F.Lara, Garilleti & Goffinet, *L. leptocarpa* (Bruch & Schimp. ex Müll.Hal.) Vigalondo, F.Lara & Garilleti, *L. affinis* (Brid.) F.Lara, Garilleti & Goffinet, and *L. fastigiata* (Brid.) Vigalondo, F.Lara & Garilleti. On the other hand, the clade corresponding to group B (including morphotypes 2, 3, and 4) is closely related (BS=97) to *L. hookeri* (Wilson ex Mitt.) F.Lara, Garilleti & Goffinet, *L. arborescens* (Thér. & Naveau) F.Lara, Garilleti & Goffinet, and *L. shawii* (Wilson) F.Lara, Garilleti & Goffinet. The latter species is recovered as sister to group B with BS=100. Within this group B, the samples corresponding to morphotype 3 are gathered in a well-supported monophyletic group (BS=100). The rest of the samples (corresponding to morphotypes 2 and 4) form a basal evolutionary grade, with samples ascribed to morphotype 2 recovered in earlier-diverging positions, while those of morphotype 4 are closely related to morphotype 3 (BS=74). Computing splits network recovers similar results, with specimens corresponding to morphotype 1 separated in a well-defined branch, and specimens corresponding to morphotypes 2-4 gathered in another branch and showing potential phylogenetic conflicts. Within the latter group, samples of morphotype 3 are grouped in a well-defined branch.

## 4 Discussion

The biogeographic connection between East Africa and Asia, triggered by Cenozoic events, has been studied in several groups of organisms both of flora and fauna (Datta-Roy and Praveen Karanth, 2009; Karanth, 2021; Sidharthan and Karanth, 2021; Johnson et al., 2022), but rarely in bryophytes (Pócs, 1976). The case study here presented shows that such biogeographic connection is in some cases real. However, our results indicate a complex evolutionary and biogeographic scenario involving different taxonomic entities that are otherwise morphologically similar.

The findings of this study suggest that the current concept of *Lewinskya firma* encompasses distinct species that were mistakenly classified as a single entity, being an example of cryptic diversity as defined by Bickford et al. (2007). The results of both the morphological and the molecular analyses support the existence of at least two distinct taxa at the species level, since morphotype 1 is clearly differentiated from morphotypes 2, 3 and 4.

Lewinsky (1980) already discussed the similitudes of the Indian and African specimens of *Lewinskya firma*, based on the presence of samples with channeled leaf apex and sporophytes with a tendency to have eight pairs (instead of sixteen) of exostome teeth in both continents. According to our results, these traits are part of those characterizing the specimens belonging to group B, and are different to those exhibited by specimens from group A. Thus, this study indicates that Lewinsky (1980) was right in considering that the Indian populations were identical to some of the African ones, although our results reveal that not all the African populations share the same morphological features.

According to their genetic and morphological differentiation, the groups A and B represent two unambiguously distinct species, which we will hereafter refer to as *Lewinskya firma* (which corresponds to group A), and *Lewinskya afroindica* (corresponding to group B) (see Taxonomy section). Despite their superficial similarity, both species are easily distinguishable by morphological features from the gametophyte and the sporophyte (**Table 1**). Leaf apex offers the best character to distinguish both species under the stereoscopic microscope, since whereas *L. firma* has more or less acute and plane apices, *L. afroindica* shows characteristic cuspidate and channeled apices. The shape of leaf apex has turned out to be a key character for the resolution of other complexes of species within Orthotrichaceae (see, for example, Medina et al., 2013; Vigalondo et al., 2020). Other characters, such as the vaginula pilosity, have been questioned by some authors (Plášek and Sawicki, 2010). In the current case study, even if the variation in the number of hairs is not conclusive to separate both species, the shape and papillosity of the hairs can be safely used. Thus, in *L. firma* the vaginula is typically naked or with few slightly papillose hairs, whereas in *L. afroindica* the vaginula is always more or less hairy and the hairs show prominent papillae and even cells that stand out forming teeth. However, the most suitable character to distinguish between both species under the microscope is the spore ornamentation. While *L. firma* shows smooth or slightly rugulose spores, in *L. afroindica* the spores are distinctly papillose. The ornamentation of the spores is an important character in the taxonomy of Orthotrichaceae, as it is in several other bryophyte groups (Goffinet and Shaw, 2009), and smooth spores are exceptional among Orthotricheae (Lewinsky, 1993), the tribe where *Lewinskya* belongs.

**Figure 3:**
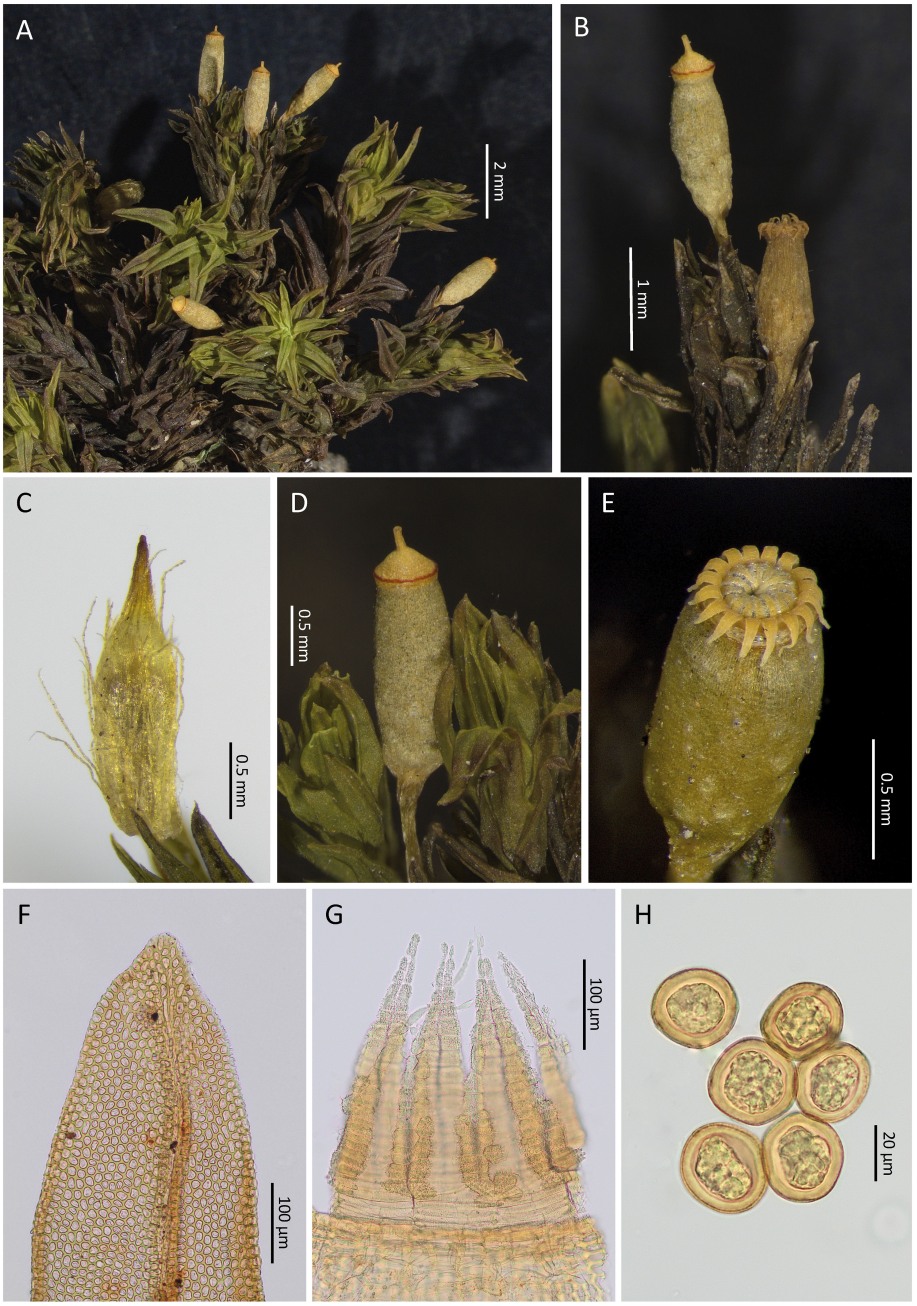
*Lewinskya firma*. **(A)** Habit. **(B)**, Detail of two capsules in different stages of development. **(C)**, Calyptra. **(D)**, Operculate capsule with a conspicuous thin red basal ring in the lid. **(E)**, A typical peristome when dry, with 16 recurved exostome teeth and 16 incurved endostome segments; note the hyaline and shine layer connecting the endostome segments. **(F)**, vegetative leaf apex. **(G)**, Detail of the peristome; the endostome segments seem not to be connected to the capsule mouth since their basal parts are hyaline and lack any ornamentation. **(H)**, Spores. Source of images: **(A, C, D)**, MAUAM 3632; **(B, E, G, H)**, MAUAM 3670; **(F)**, MAUAM 3623. Photographs by R. Garilleti & R. D. San Román.

**Figure 4:**
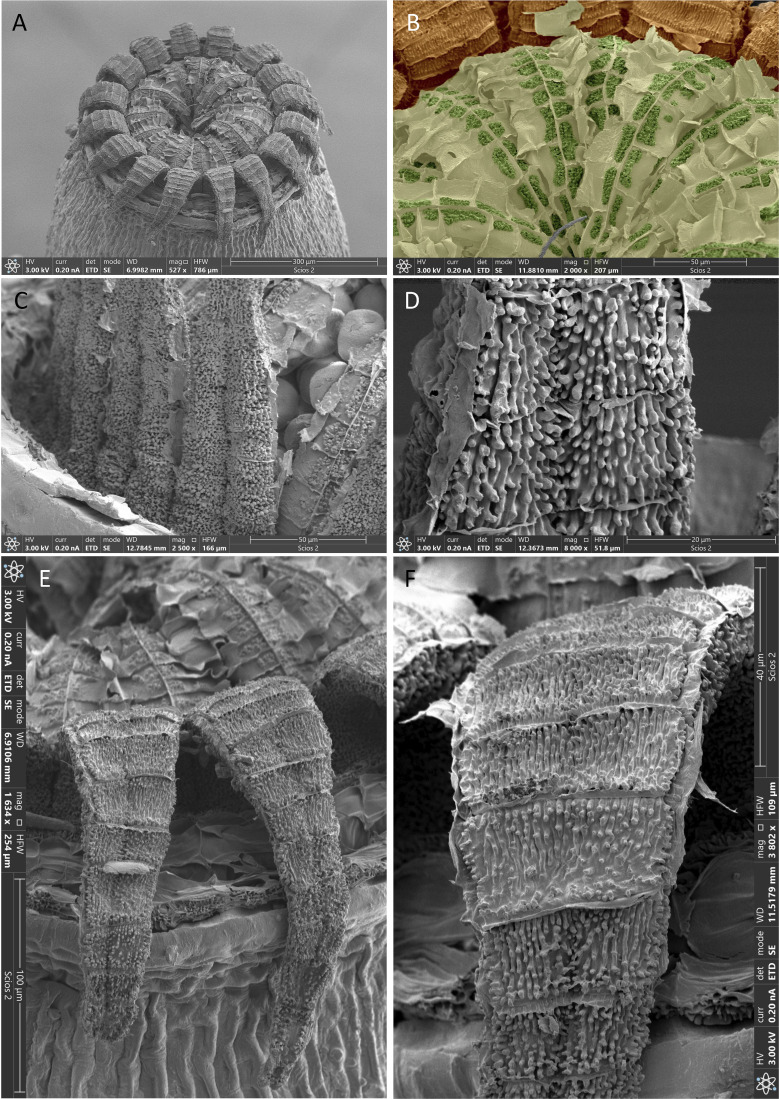
*Lewinskya firma*, SEM micrographs. **(A)** General view of peristome. **(B)** Endostome external side (endostome Primary Peristomial Layer, PPL) in false color, where it can be seen that it is a continuous layer (light green) extending both on the segments and over the space between them; each segments can be identified by a median vertical line separating two rows of cell areas, most of which are partially ornamented with papillae (highlighted in dark green); the layer (PPL) between the segments have no papillae although its cell areas are clearly delimited by horizontal lamellae; in the upper part of the picture, the inner side (exostome Primary Peristome Layer) of the basal portion of some exostome teeth (in brown) can be seen. **(C)** External side of exostome (OPL, Outer Peristomial Layer); note the almost smooth spores in the background. **(D)** Detail of the OPL ornamentation. **(E)** General view of two recurved exostome teeth; the endostome can be seen in the upper part of the image. **(F)** Detail of the ornamentation of exostome internal side (exostome Primary Peristome Layer). Source of images: **(A–C, F)**, MAUAM 3670; **(D, E)**, MAUAM 3650. Photographs by R. Garilleti.

**Figure 5:**
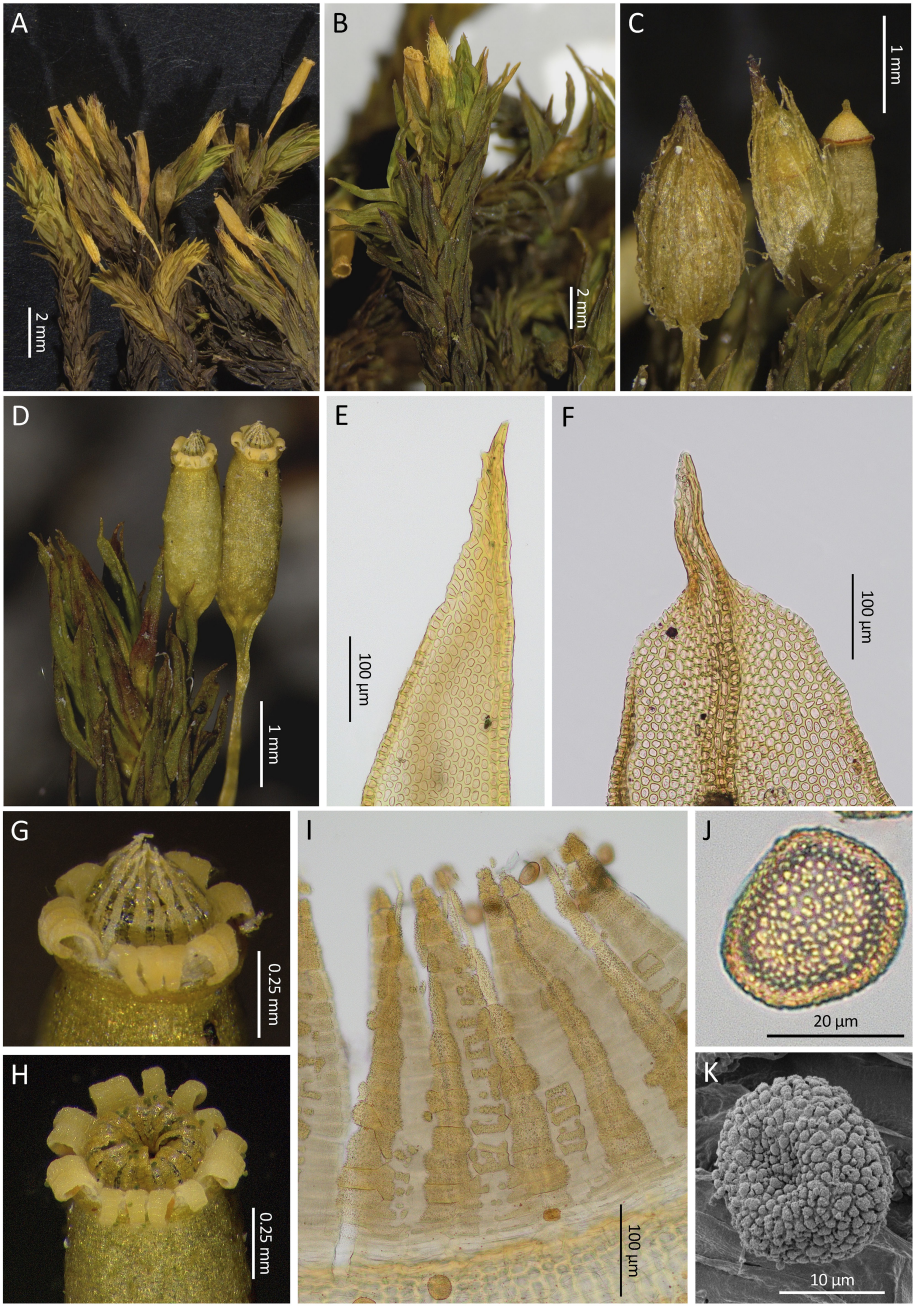
*Lewinskya afroindica*. **(A)** Habit of a specimen with exserted capsules. **(B)** Detail of the habit of a specimen with immersed to hemiemergent capsules. **(C)** Calyptrae and an operculate capsule. **(D)** Two mature capsules. **(E, F)** Variability in the apices of vegetative leaves. **(G, H)** Aspect of peristomes when dry, with the revolute exostome teeth in 8 pairs, some of them split, and the endostome segments erect or incurved; the hyaline layer connecting the segments can be seen in both cases thanks to its brightness. **(I)** Detail of the peristome from the inside showing in the foreground the Inner Peristome Layer (IPL) which is densely papillose; the IPL develops along the segments and in a fragmentary manner, irregularly pectinate, in the spaces between segments; the endostome Primary Peristome Layer (PPL) is not visible, because although continuous it is a thin and transparent layer that is only partially ornamented in some central areas of the segments, being hidden by the papillosity of the IPL. **(J)** LM image of a spore. **(K)** SEM image of a spore. Source of images: **(A)**, MAUAM 3628; **(B)**, MAUAM 3627; **(C)**, MAUAM 5590; **(D, G)**, MAUAM 5603; **(E)**, MAUAM 3643; **(F)**, MAUAM 5584; **(H, J)**, MAUAM 3647; **(I)**, MAUAM 3638; **(K)**, MAUAM 5600. Photographs by R. Garilleti & R. D. San Román.

**Figure 6:**
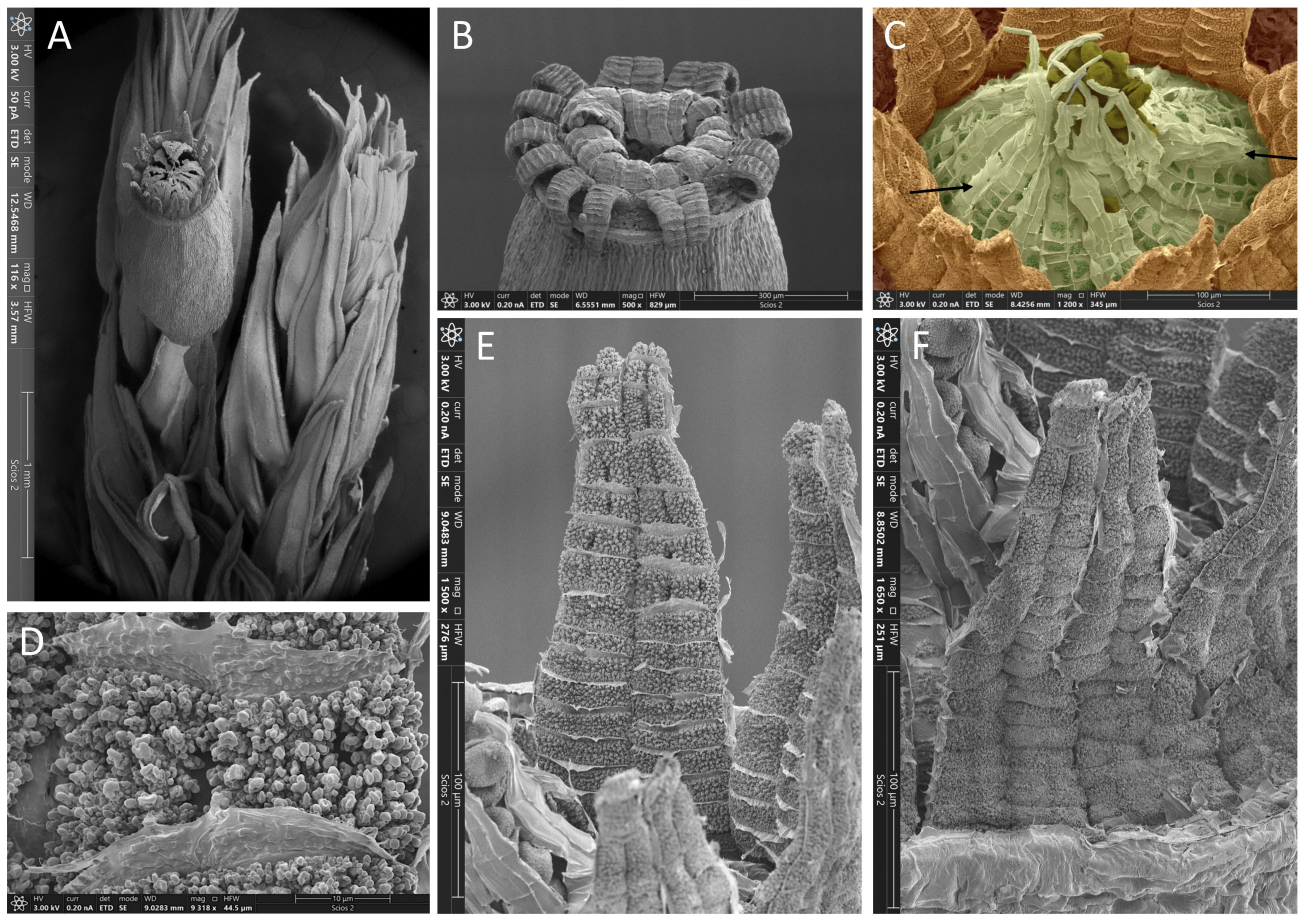
*Lewinskya afroindica*, SEM micrographs. **(A)** Upper part of a gametophore with one capsule; note the long cuspidate apices of the upper leaves and the faintly ribbed upper part of the capsule. **(B)** General view of the peristome. **(C)** Detail of a peristome in false color, centered in the endostome (light green); only the external side (Primary Peristomial Layer, PPL) of the endostome can be seen, with the segments traversed by a median vertical line from which transversal lines delimit two rows of cell areas, some of which are partially ornamented with papillae (highlighted in dark green); this layer (PPL) also extends between the segments although it is only visible in some spaces where it is not folded (arrows), being the cellular areas barely marked and devoid of ornamentation by papillae; the exostome teeth (in brown) have become semi-erect after removal of the operculum; the spores (dark olive green) remain retained by the tips of the segments in this initial phase of the spore release. **(D)** Detail of the ornamentation of the exostome internal side (exostome Primary Peristome Layer). **(E)** General view of the internal side (exostome PPL) of two paired teeth. **(F)** External side (OPL, Outer Peristome Layer) of two paired teeth. Source of images: **(A, B)**, MAUAM 3647; **(C–F)**, MAUAM 5585. Photographs by R. Garilleti.

We also found subtle differences in the peristome characteristics. The two species have apparently the same peristome constitution, with 16 exostome teeth and 16 wide endostome segments. This configuration is very uncommon within the genus, and no other species in sub-Saharan Africa exhibits it (Lewinsky, 1978, 1993), which could be one of the reasons explaining the previous taxonomic treatment as a single species. In the present study, we have found that the constitution of the endostome of the two species is very original, since the segments are initially joined by a thin and fragile coating that occludes a great part of the mouth of the capsule until it is disintegrated (**Figures 3E**, **4A, B**, **5G, H**, **6B, C**). However, we have also found peristomial differences between the two species, affecting both the constitution of the exostome teeth, their ornamentation, the way they are recurved when dry, and the constitution and ornamentation of the endostome. The exostome in *L. firma* has 16 independent teeth that are separated from the initial stages, ornamented with thick and high papillae, and generally recurved against the capsule exothecium. In *L. afroindica*, the exostome is originally formed by 8 teeth pairs, but these easily split with the first hygroscopic movements, although frequently some of these pairs persist; moreover, the teeth are covered by granulose papillae, and are revolute when dry, only the point touching the capsule exothecium. As for the endostome, segments in *L. firma* seem to develop at a certain distance from the capsule mouth, since they arise from a tall connective membrane that is thin, hyaline and smooth, and the segments are scarcely ornamented in the external surface; in contrast, in *L. afroindica*, segments seem to develop near the capsule mouth, since the connective membrane is lower, and they are neatly ornamented on the external surface.

Conversely, seta length does not seem to be a desirable character to separate the two species, although it can be diagnostic in other Orthotrichaceae (eg. Matanov et al., 2024). In both groups A and B, high variability has been found in seta length, with several specimens showing shortly exserted to almost emergent capsules whereas others present long exserted capsules. This is especially evident in group B because specimens from disjunct populations show extreme variability of seta length, which initially made us consider the possible existence of separate morphotypes. However, variation in seta length has been found in each of the disjunct areas studied for group B. Thus, even if the overlapping measures do not allow for a segregation based on this character, in Eastern Africa seta length shows a cline variation, with the lower values (corresponding to morphotype 2) in the northern limit of the group B geographic area, the larger values (corresponding to morphotype 4) in the southern limit, and specimens belonging to one or another morphotype in a wide central area. In the case of India (morphotype 3), setae are also generally long. Moreover, although the molecular results do not clearly support the hypothesis of four different taxa corresponding to the four morphotypes, they are compatible with it, and in the case of the Indian specimens, genetic data support a certain degree of differentiation.

In line with the morphological differences here exposed, our results based on molecular data support that *L. firma* and *L. afroindica* constitute two separate species. A preliminary study based on Sanger sequencing of a larger representation of *Lewinskya* species also suggested genetic variation among these two taxa, although low resolution in the overall tree was displayed (results unpublished). Sanger sequencing usually provides low resolution to resolve phylogenetic relationships within *Lewinskya* (e.g. Draper et al., 2021), and such lack of resolution has been found even when using target enrichment approaches (Draper et al., 2022). For this reason, we selected GBS, which is generally effective at the level of closely-related populations and species (Elshire et al., 2011), and that is also useful at upper taxonomic levels (Hamon et al., 2017; Martín-Hernanz et al., 2019). With this methodology, the specimens corresponding to *L. firma* and *L. afroindica* are grouped in fully supported (PP=100) clades that are not phylogenetically closely related (**Figure 2**). The lack of support for some of the nodes recovered, possibly resulting from potential phylogenetic conflicts as shown in the splits network, as well as the incomplete phylogenetic frame for the genus, precludes definitive conclusions on the relationships of *L. firma* and *L. afroindica* with other species. Even though all the African species of *Lewinskya* were included in the study, the recovered groups do not show a clear geographic pattern, since the African species appear scattered in the two main clades. On the one hand, *L. firma* seems to be related to the orophilous African endemic *L. tanganyikae* and to other African species of this genus (*L. galiciae*, *L. leptocarpa*), but also to species such as *L. graphiomitria* (from New Zealand) or the Mediterranean *L. affinis* and *L. fastigiata*. On the other hand, *Lewinskya afroindica* is related with full support to the Mediterranean *L. shawii*, and both are included in a well-supported clade with the African species *L. arborescens*, as well as with one Himalayan species (*L. hookeri*).

We did not identify morphological patterns that could explain the obtained phylogenetic relationships. As an example, the peristomial constitution of *L. firma* and *L. afroindica* shows characteristics that are unique in the genus, such as the fragile thin coating that initially connects the segments. However, neither this nor other morphological features shared by both taxa seem to be the result of recent common ancestry. Moreover, *L. arborescens*, the most similar species to *L. afroindica* and *L. firma* based on its exserted capsules and peristomial configuration, is not recovered as sister to any of the two. In this scenario, the similarities in morphology are most likely the result of convergent evolution (homoplasy), similar to what has been reported for several genera of Orthotrichoideae (Draper et al., 2021).

It seems clear that further phylogenetic analyses are needed to understand the relationships within *Lewinskya*, including those of *L. firma* and *L. afroindica*. A wider representation of the genus is needed, but also finding another source of molecular data, given the problems of resolution in phylogenies based on Sanger (e.g. Draper et al., 2021), target enrichment approaches (Draper et al., 2022), and GBS (this study). In any case, the present work provides clear evidence on the genetic distance between *L. firma* and *L. afroindica* and their segregation at the species level. Similar cases of unexpectedly high genetic distance unmasked by morphological similarity have been previously reported in Orthotrichaceae (e.g. Medina et al., 2013), and could be explained by homoplasy related to habitat adaptation (Draper et al., 2021).

In addition, the present study reveals a probable incipient speciation pattern within *L. afroindica*. The clade corresponding to *L. afroindica* (group B in **Figure 2**) gathers samples initially ascribed to three different morphotypes, namely 2, 3 and 4. Neither morphotype 2 nor morphotype 4 constitute monophyletic groups, but samples of morphotype 3, all of them from India, are grouped in a fully supported clade (PP=100) with a relatively long branch at the base, which is also recovered in the splits network. So far, we have failed finding a set of diagnostic morphological characters to undoubtedly identify samples of this morphotype, and we therefore currently refrain from proposing its segregation as a separate species. In any case, our results suggest that this could be a case of incipient nested speciation (Crawford, 2010). This type of speciation, in which a population becomes adapted to a new habitat and splits from a progenitor species, whereas the ancestral species remains more or less unchanged in its original habitat, has been previously reported for several groups of organisms, including vertebrate animals (e. g. Kuchta et al., 2018), several invertebrates such as Orthoptera (e. g. Kaya and Çiplak, 2016), and many groups of plants (e. g. Otero et al., 2022). There are similar cases of incipient speciation where the derivative taxa are recognized at the species level; see, for example, Alonso et al. (2022) for an illustration in the angiosperm family Araliaceae Juss., or Matanov et al. (2024) for such a case in Orthotrichaceae. In all these examples, the incipient genetic segregation encompasses a phenotypic differentiation that remains constant. However, as indicated above, in our study the differentiating morphological characters found in samples ascribed to morphotype 3 are embedded within the variation observed in the African populations of *L*. *afroindica* (morphotypes 2 and 4). The isolated distribution of the samples of morphotype 3 in India suggests that the ongoing segregation process will eventually lead to a separate species. However, it seems more sensible to consider that currently there is one single species, due to the lack of a clear phenotypic variation, the close genetic relationship of the Indian and African populations, and the existence of potential phylogenetic conflicts.

Our taxonomic solution raises an interesting biogeographic question, concerning the possible origin of the disjunct distribution between Africa and India. The monophyly of the samples from India in a separate group nested within the African populations suggests a probable scenario of a single event of colonization from southern or eastern Africa (excluding the nearest Ethiopia) to India. The geographic isolation has probably prevented further genetic exchange. Given the genetic proximity between the Indian and African populations, the colonization event probably involved long-distance dispersal, rather than vicariance, since the separation of the Indian subcontinent and Africa dates back to the Mesozoic (Upchurch, 2008), and it is unlikely that there was a continuous ancient distribution from Africa to India that later fragmented. Both the crown age of the genus *Lewinskya* around the Oligocene (Draper et al., 2021) and the fact that we have found no clear morphological differentiation between the populations of the two continents suggest that the colonization event is recent.

This is a very rare disjunction among those affecting Africa and Asia, which generally involve species more widely distributed on both continents and not restricted to India in Asia (Pócs, 1976). In India, *L. afroindica* has been found at two separate mountain ranges at elevations between 2100 and 2400 m a.s.l. This indicates that the population there is stabilized and that after the initial colonization this species probably spread to other suitable habitats in the southern Indian mountains.

## 5 Taxonomy

The presence in East Africa of two different taxonomic entities that were previously considered to be a single species, *Lewinskya firma*, makes it necessary to determine which of these entities aligns more closely with the original concept of its author, in order to maintain nomenclatural stability. The immediate step would be to compare with the original material used by Venturi (1872) to describe *Orthotrichum firmum* Venturi, collected by O. Beccari near Keren in the Eritrean Anseba region, referred to in the protologue as “Bogos” after the dominant ethnic group in the area. However, it has been impossible to study these specimens, which, like the rest of his herbarium, should be in TR. This specific material has been sought on several occasions (Lewinsky, 1978; Vitt and Nimis, 1987; Garilleti et al., 2007) without success, and is currently considered lost. On the other hand, Dixon (1922) attributes the collection of the type of this species to G.W. Schimper, a collector of exsiccatae, from which the potential existence of duplicates to study this species could be inferred. However, this option was discarded since Cufodontis (1951) and Gillet (1972), demonstrated that Schimper’s botanical explorations in Ethiopia and Eritrea did not include the Anseba region.

Nevertheless, in the protologue of *O. firmum* (Venturi, 1872) certain characters can be identified, allowing an unequivocal assignment of his species to one of the morphotypes recognized in the present study. The most relevant, being eminently qualitative, is the ornamentation of the spores, which are practically smooth in *O. firmum* (‘*sporae virides laevissimae*’, **Figures 3H**, **4C**), but also noteworthy is the slightly hairy calyptra in Venturi’s species (‘*calyptra immatura basi tantum parum pilosa*’, **Figure 3C**). These characters are found in morphotype 1, while the spores of the other morphotypes are densely papillose (**Figures 5J, K**) and the calyptras typically densely hairy (**Figure 5C**). The position of peristomial teeth, another qualitative differential character among morphotypes, cannot be employed because the material studied by Venturi was damaged, and this author could not confidently establish the actual position of the teeth in a dry state (‘*ex fragmentulo peristomatis directionem dentium externorum videre nequivi, sed revolutos esse credo*’, ‘I could not see the orientation of the external teeth from the small portion of the peristome, but I believe they are revolute’). As a conclusion, morphotype 1 seems to fully coincide with Venturi’s concept of the species, so the name *Lewinskya firma* applies to it.

Once the morphotype that aligns with Venturi’s original concept of the species has been identified and in the absence of original material, it is necessary to select a neotype according to Art. 9.8 of the International Code of Nomenclature (Turland et al., 2018). The specimen F. Lara 1311/064 has been chosen due to its abundance, completeness, and high representativeness of the species.

### 5.1 Species descriptions

***Lewinskya firma*** (Venturi) F.Lara, Garilleti & Goffinet in Lara et al., Cryptogamie, Bryologie 37(4): 374 (2016) (**Figures 3**, **4**).

≡*Orthotrichum firmum* Venturi, Nuovo Giornale Botanico Italiano 4: 15. (1872). Type: Bogos Abyssiniae circa Keren, *Beccari s.n.*, (not found, originally in TR, probably lost), Neotype (designed here): ETIOPÍA: Āmara, N. Gonder, Simien Mts., Gimbar river on the way from Ayna Medda camp to Guinch camp, 13°15’22”N, 38°06’42”E., 3316 m, branches of *Erica*. Shrubby bushes of heather (*Erica trimera*) and *Hypericum*, 18/11/2013, *F. Lara 1311/064 with V. Mazimpaka & B. Vigalondo.* (BM; Isoneotype, MAUAM 3670).

Plants 1.0–2.5 cm tall, in lax or rarely dense cushions, olive green turning to brownish below. Stems rounded in section; axillary hairs 4–6 cells long, 1–2 short colored basal cells and (2–)3–4(–5) elongated and hyaline distal cells. Rhizoids restricted to basal parts of stems, reddish, smooth. Leaves densely arranged, erect-appressed to erect-patent when dry, erect-patent to spreading when moist, lanceolate to ovate-lanceolate, keeled, not undulate, 2.3–4.2 × 0.6–1.1 mm; leaf apex acute, short acuminate or ending in a mucro; costa percurrent, 60–70 μm wide along leaf, orangish; leaf margins recurved to revolute on both sides, plane near apex, entire except at base, where it is crenulate-papillose; lamina unistratose throughout; upper and median leaf cells ovate to isodiametric, sometimes oblate, (8–)11–16(–20) × (7–)9–13 μm, with (1–)2(–3) tall, blunt papillae; basal cells rectangular, (11–)16–25(–33) × 7–11 μm, with thickened, straight or slightly undulate walls, rarely with intermixed very short rows of colored nodulose cells; basal paracostal cells rectangular to trapezoid, 36–70(–75) × (8–)11–16 µm, with thick nodulose or more rarely straight cell-walls, smooth or with 1–2 very low papillae; basal marginal cells in 6–12 rows of short rectangular to quadrate cells, (10–)14–27(–38) × (9–)10–15(–17) μm, with cell walls straight, evenly thickened, smooth or with 2 papillae on cell extremes; small auriculae occasionally differentiated, with large coloured cells.

Gonioautoicous. Perigonial leaves ovate, with apex acute to acuminate, entire, green to brown. Perichaetial leaves not differentiated. Vaginula short cylindric, naked, occasionally with 1 to several long papillose hairs, exceptionally hairier; hairs short, hyaline or yellowish, 1–2 seriate, thin to ± thickened walled, smooth to slightly papillose. Calyptra conic-oblong, 2.5–3.0 mm long, plicate to almost smooth, completely covering capsule, yellowish with a dark orange beak, openly to occasionally densely hairy, with long, thin, moderately papillose hairs, more abundant towards base, frequently overpassing base of beak. Polysety very rare, with 2 sporophyte per perichaetium. Seta 1.1–3.4 mm long, straight or lightly counterclockwise twisted when dry, smooth, yellowish. Capsule usually short exserted, occasionally hemi-emergent, rarely long exserted, ovate-cylindrical, cylindrical or fusiform when dry, ovate when wet, 1.3–2.0 mm long, smooth, cream-colored when mature turning pale brown in old capsules, with short neck, gradually contracted to seta; exothecial cells rectangular to trapezoid; suboral cells in 4–5 rows of oblate cells with thickened walls; exothecial bands not differentiated. Stomata located in lower 2/3 of capsule, rarely reaching neck. Operculum rostrate, conic to convex with a thin reddish basal rim, 0.65–0.7 mm in diameter. Peristome double; prostome occasionally seen, fragile, deciduous, initially formed by several rows of cells, the basal one papillose, the others smooth; exostome teeth 16, independent from the beginning, yellowish, 300–380(–410) μm long, reflexed and leaning against the exothecium when dry, occasionally revolute touching the exothecium by tip; outer peristome layer (OPL) ornamented with a papillose reticulum, papillae tall, thick, simple or ramified; exostomial primary peristome layer (PPL) with longitudinal thick papillose lines, trabeculae occasional, thin, tall and smooth; endostome initially almost continuous, with a tall connecting membrane from which 16 wide segments arise, joined in most of their length by a fragile, shiny hyaline coating; connecting membrane with 4–6 rows of rectangular, hyaline and smooth cell areas, somewhat exceeding capsule mouth, being barely perceptible under light microscope; segments lanceolate, ornamented, yellowish, as long or slightly longer than teeth; endostomial PPL continuous in lower 2/3, on segments ornamented with papillose patches within cell areas near segment keel, between segments smooth or with sparse papillae; lamellae well developed, thin, tall and smooth delimiting cell areas of both segments and intersegment surfaces; inner peristome layer (IPL) densely papillose, with papillae tall, thick and ramified, trabeculae thin, ± tall and smooth. Spores (22–)26–33 μm, smooth to very finely papillose, almost inconspicuous, rugulose, isodiametric, yellowish brown.

Distribution. *Lewinskya firma* is a widely distributed moss in the highlands of Ethiopia, extending southwards through the volcanic mountains of the Great Rift. Our collections report it between 1935 and 3765 m elevation. It is particularly abundant in the Simien Mountains in northern Ethiopia, located about 250 km south of its original description site in the central mountains of Eritrea. Additionally, it is also very common in the Bale Mountains, southwest of the Great Rift in Ethiopia. In the sampling conducted for this study, *L. firma* was also discovered in Mount Kenya (Kenya) and, already in the Southern Hemisphere, in Mount Kilimanjaro (Tanzania), as well as in the Ngorongoro region (Tanzania), and in the northern volcanoes of Rwanda. It is highly likely that it also inhabits other ‘sky islands’ within the Afromontane and Afroalpine habitats of the region. A throughout review of herbarium specimens identified as *Orthotrichum firmum* would be necessary to precisely determine the actual distributional area of this species, particularly with regards to its southern limit.

***Lewinskya afroindica*** F.Lara, Garilleti & Draper sp. nov. **Figures 5**, **6**.

Type: INDIA: Tamil Nadu, Dindigul Dist., Kodaikanal, Kodaikanal Lake, Lake Rd, 10°14’00” N, 077°29’10” E, 2100 m, on trunk of *Alnus* sp., 3/1/2019, *F. Lara & J. Lara 1901/12* (Holotype at BM; Isotypes MAUAM 3647, MO).

Diagnosis: Similar to *Lewinskya firma*, from which it differs mainly in the often-cuspidate leaves with channeled tips, the hairy vaginula, with strongly papillose or toothed hairs, and the strongly papillose spores.

Plants (0.4–)1.1–3.0(–4.6) cm tall, in lax or dense cushions, olive green turning to brownish below. Stems rounded in section; axillary hairs 3–7 cells long, 1–2 short colored basal cells and (2–)3–4(–6) elongated and hyaline distal cells. Rhizoids in basal parts of stems, reddish, smooth. Leaves densely arranged, erect-appressed to patent when dry, erect-patent to spreading when moist, lanceolate to ovate-lanceolate, keeled, sometimes ± undulate, (2.2–)3.0–4.5(–5.0) × (0.6–)0.7–1.2(–1.4) mm; leaf apex acuminate or cuspidate, the point variably long and frequently channeled, acute or truncate, occasionally ± dentate; costa percurrent, 50–80(–97) μm wide along leaf, usually orangish below and greenish in upper half; leaf margins recurved to revolute in most length, frequently one margin more strongly so, entire in upper half, often papillose bellow; lamina unistratose throughout; upper and median leaf cells ovate to isodiametric, sometimes oblate, (8–)9–22(–28) × 8–17(–25) µm, slightly papillose, with 1–2(–3) low, simple and blunt, rarely slightly ramified, papillae; basal cells rectangular, (10–)15–25(–50) × 11–15 μm, with thickened, straight or slightly undulate walls, smooth, prorate or with distal and proximal prominent papillae, especially in upper basal zone, rarely with intermixed short or long rows of colored nodulose cells; basal paracostal cells rectangular, 30–70(–92) × (6–)10–15(–21) µm, with thin to moderately thickened, straight or nodulose cell-walls; basal marginal cells in 6–12 rows of short rectangular to quadrate cells, (8–)11–22(–35) × (10–)11–16(–23) µm, with cell walls straight, thickened, especially transversal ones, often with prominent papillae in distal and proximal ends, becoming geminate at margins; auriculae occasionally differentiated, slightly prominent at margin, with some enlarged cells.

Gonioautoicous. Perigonial leaves ovate, with apex obtuse to acuminate, entire or occasionally papillose-dentate, brown with green tips. Perichaetial leaves not differentiated, long channeled-cuspidate similarly to other upper leaves. Vaginula cylindric to doliform, hairy, with ± numerous hairs, rarely scarce; hairs short, hyaline, 1–3 seriate, thin to slightly thickened walled, strongly papillose, and typically with some projecting cells forming straight or retrorse teeth, occasionally also with more or less numerous long, pluriseriate, yellowish hairs. Calyptra conic, 2.2–3.6 mm long, plicate, completely covering capsule, yellowish with dark beak, densely or rarely openly hairy; hairs short, no overpassing base of beak, thin, pluriseriate, papillose, occasionally also dentate. Polysety occasional, with 2(–3) sporophyte per perichaetium. Seta (0.8–)1.1–4.0(–4.7) mm long, straight or counterclockwise twisted when dry, smooth, yellowish. Capsule short to long exserted, occasionally hemi-emergent, ovate to ovate-cylindrical when dry, ovate when wet, (1.2–)1.5–2.5 mm long, smooth, rarely faintly ribbed in upper part, cream-colored when mature turning pale brown in old capsules, with short neck, gradually contracted to seta; exothecial cells rectangular to trapezoid; suboral cells in 3(–5) rows, oblate, with thickened walls; exothecial bands not or hardly differentiated. Stomata located in lower 1/2–2/3 portion of urn. Operculum rostellate, conic to convex, cream-orangish, with a thin reddish basal rim, (0.6–)0.7–0.9(–1.0) mm in diameter. Peristome double; prostome rarely seen, incomplete, low, papillose; exostome teeth 16, originally in 8 pairs that quickly split, some pairs often remain partially united after hygroscopic movements; teeth cream to orange colored, (225–)270–450 µm long, revolute when dry, touching the exothecium by tip, occasionally recurved, fragile, often broken after recurving; outer peristome layer (OPL) very densely ornamented with granulose papillae, almost hiding basal reticulum; exostomial primary peristome layer (PPL) densely covered with tall, ramified and granulose papillae, often arranged in longitudinal lines bellow, with thin, tall trabeculae; endostome initially almost continuous, at base with a connecting membrane from which 16 wide segments arise, joined in most of their length by a fragile, shiny hyaline coating; connecting membrane with 2–3 rows of rectangular, hyaline and smooth cell areas, somewhat exceeding capsule mouth, being barely perceptible under light microscope; segments lanceolate, ornamented, yellowish to orangish, as long or slightly longer than teeth; endostomial PPL continuous in lower 2/3, on segments ornamented with irregular papillose patches in some cell areas near segment keel, between segments smooth to rugulose; lamellae delimiting cell areas smooth and low, only on segments; inner peristome layer (IPL) densely papillose, papillae tall, simple, covering segments and appendages between segments, which often are numerous and form pectinate structures; thin trabeculae occasional. Spores (18–)21–28(–35) µm in diameter, strongly papillose, thick walled, spherical to ellipsoid, yellowish brown to green.

Distribution. *Lewinskya afroindica* exhibits a broad disjunct distribution across territories surrounding the Western Indian Ocean. In Africa, it has been found in the highlands of Ethiopia, Kenya, Tanzania, Rwanda, and South Africa. We have found it between 2210 and 3900 m elevation, although in South Africa it occurs from 1225 m upwards. It is presumed to inhabit most, if not all, of the ‘sky islands’ within the Afromontane and Afroalpine regions, situated above (1200–)2000 m.a.s.l. In India, it is found in at least two areas near the Southern Western Ghats. Apart from the nucleus where it was initially identified in the Nilgiri Hills (Lewinsky, 1980), a second nucleus has been discovered approximately 100 km to the south, in the Palani Hills. In both cases, *L. afroindica* thrives above 2100 m.a.s.l. To date, all Indian collections have been conducted in somewhat anthropized environments, mainly urban areas of Udhagamandalam and Kodaikanal. However, it is highly probable that populations exist in more natural settings within these regions.

## Data Availability

The datasets presented in this study can be found in online repositories. The names of the repository/repositories and accession number(s) can be found below: https://www.ncbi.nlm.nih.gov/, Bioproject PRJNA1134949.
