# Supplementary material for: Unveiling the diversification and dispersal of the Lewinskya firma complex (Orthotrichaceae, Bryophyta) across Africa and India
Source: Front Plant Sci. 2024 Sep 27;15:1451005. doi: 10.3389/fpls.2024.1451005 (PMC11466771; doi:10.3389/fpls.2024.1451005)
Supplement: Supplementary file 1 [file DataSheet1.docx]

**SUPPLEMENTARY MATERIAL:**

**Annex I. Selected specimens studied**

**Annex 2.** **Samples included in the GBS analyses and their corresponding barcodes. Information regarding the number of loci recovered and the missing data percentage is included.**

**Annex I. Selected specimens studied**

***Lewinskya firma***

**ETHIOPIA**. **Oromiya**, Bale, Bale Mts, road from Dodola to Dinsho, 07º01'51" N, 039º33'39" E, 3050 m, on trunk of *Olea* sp., 6/11/2013, *F. Lara et al.* *1311/05* (MAUAM 3623); *ibidem*, on *Rhododendron* sp., 6/11/2013, *V. Mazimpaka et al. s.n.* (MAUAM 5555); Bale, Bale Mts, road from Dodola to Dinsho, 07º02'04'' N, 039º34'30'' E, 3173 m, epiphytic in juniper forest (*Juniperus procera*), 6/11/2013, *B. Vigalondo et al.* *s.n.* (MAUAM 5554); Bale, Bale Mts, near Goba towards Sanetti Plateau, 06º57'45" N, 039º57'39" E, 2933 m, on branches and twigs on thorny bush, 8/11/2013, *B. Vigalondo et al.* *s.n.* (MAUAM 5557); Jimma, Road from Jimma to Bedele, just south of Sek'a, 07º46'06" N, 036º43'42" E, 1935 m, thin trunks of coffee trees (*Coffea arabica*), 12/11/2013, *F. Lara et al.* *1311/41* (MAUAM 3741); Ilubabor, Road from Jimma to Bedele, about 25 km SE of Bedele, 08º14'46" N, 036º26'49" E, 2250 m, on trunk of a small tree, 12/11/2013, *B. Vigalondo et al.* *s.n.* (MAUAM 5558). **Amhara**, Agew Awi, Road from Bure to Bahir Dar, about 4 Km south of Adis Kidame, 11º02'44" N, 036º54'06" E, 2555 m, on *Acacia* sp., 13/11/2013, *B. Vigalondo et al.* *s.n.* (MAUAM 5560); N. Gonder, Escarpment of the Simien Mts. north of Debark, about 7 km on the road to Chew Ber, 13º11'38" N, 037º53'23" E, 2820 m, on trunk of *Rosa abyssinica*, 16/11/2013, *F. Lara et al.* *1311/48* (MAUAM 5561); N. Gonder, Escarpment of the Simien Mts. north of Debark, about 10 km on the road to Chew Ber, 13º11'49" N, 037º53'14" E, 2700 m, on trunk of small tree, 16/11/2013, *F. Lara et al.* *1311/50* (MAUAM 5562); N. Gonder, Simien Mts, escarpment east of Simien lodge, 13º12'39" N, 037º59'04" E, 3215 m, on trunk base of *Rosa abyssinica*, 17/11/2013, *F. Lara et al.* *1311/53* (MAUAM 5563); N. Gonder, Simien Mts, escarpment east of Simien lodge, 13º12'37" N, 037º59'08" E, 3170 m, on branches of *Rosa abyssinica*, 17/11/2013, *F. Lara et al.* *1311/55* (MAUAM 5564); N. Gonder, Simien Mts, escarpment east of Simien lodge, 13º12'13" N, 037º59'31" E, 3105 m, on branches of large shrub (Rhamnaceae), 17/11/2013, *F. Lara et al.* *1311/59* (MAUAM 5565); N. Gonder, Simien Mts, Sankaber camp, 13º13'50" N, 038º02'27" E, 3250 m, on low branches of shrubby *Olea*, 17/11/2013, *V. Mazimpaka et al. s.n.* (MAUAM 5553); N. Gonder, Simien Mts, Gimbar river on the way from Ayna Medda camp to Guinch camp, 13º15'22" N, 038º06'42" E, 3316 m, on branches of a *Hypericum* tree, 18/11/2013, *B. Vigalondo et al.* *s.n.* (MAUAM 3632, 3633); N. Gonder, Simien Mts, way from Ayna Medda camp to Guinch camp, 13º15'30" N, 038º06'41" E, 3400 m, on bent trunk of *Lobelia rhynchopetalum*, 18/11/2013*, F. Lara et al.* *1311/68* (MAUAM 5551); N. Gonder, Simien Mts, upper part of the forest below Guinch camp, 13º15'50" N, 038º06'34" E, 3515 m, on branches of *Erica* *arborea*, 18/11/2013, *B. Vigalondo et al.* *s.n.* (MAUAM 5552); Amhara, N. Gonder, Simien Mts, around Chenek Camp, 13º15'38" N, 038º11'32" E, 3595 m, on branches of a *Hypericum* tree, 19/11/2013, *F. Lara et al.* *1311/079* (MAUAM 3636, 3637); N. Gonder, Simien Mts, Chenek Camp, south slope near the camp, 13º15'22" N, 038º11'29" E, 3570 m, on trunks of *Lobelia rhynchopetalum*, 19/11/2013, *B. Vigalondo et al. s.n.* (MAUAM 5567); N. Gonder, Simien Mts, Chenek camp, escarpment ridge around viewpoint, 13º15'35" N, 038º11'23" E, 3510 m, on branches of *Erica arborea*, 19/11/2013, *B. Vigalondo et al.* *s.n.* (MAUAM 5568); N. Gonder, Simien Mts, Ambaras, Guimbar river, tributary side valley, 13º15'22" N, 038º06'45" E, 3340 m, on branches of *Erica arborea*, 20/11/2013, *F. Lara et al.* *1311/92* (MAUAM 5569 ); N. Gonder, Simien Mts, surrounding area of Jinbar waterfall, on north exposure slope, 13º14'03" N, 038º03'56" E, 3175 m, on trunk of *Erica*, 20/11/2013, *V. Mazimpaka et al. s.n.* (MAUAM 3649). **KENYA**. **Meru** **Co.**, Mt. Kenya,

***Lewinskya afroindica***

**ETHIOPIA**. **Shewa**, Addis Ababa, Addis Ababa university campus, Arat kilo, 09°01'47'' N, 038°45'53'' E, 2433 m, on *Schrebera* sp., 9/12/2008, *K. Hylander 5940* (MAUAM 4686). **Oromiya**, Bale, Bale Mts, road from Dodola to Dinsho, 07º01'51" N, 039º33'39" E, 3050 m, on tree trunk, 6/11/2013, *F. Lara et al.* *1311/06* (MAUAM 3699); Bale, Bale Mts, road from Dodola to Dinsho, 07º02'04'' N, 039º34'30'' E, 3173 m, epiphytic in juniper forest (*Juniperus procera*), 6/11/2013, *B. Vigalondo et al.* *s.n.* (MAUAM 5581); *ibidem*, branches of lauroid small tree, 6/11/2013, *F. Lara et al.* *1311/12* (MAUAM 5580); Bale, Bale Mts, Dinsho, Juniper forest (*Juniperus procera*) next to the entrance of the national park, 07º05'40" N, 039º47'35" E, 3227 m, on branches of a small tree (*Maytenus*), 7/11/2013, *F. Lara et al*. *1311/16* (MAUAM 5585); Bale, Bale Mts, Dinsho, road to the N of the national park, 07º05'08" N, 039º41'19" E, 3100 m, on isolated *Juniperus procera* trees, 7/11/2013, *B. Vigalondo et al.* *s.n.* (MAUAM 5586); Bale, Bale Mts, near Goba towards Sanetti Plateau, 06º57'45" N, 039º57'39" E, 2933 m, on branches and twigs on thorny bush, 8/11/2013, *B. Vigalondo et al.* *s.n.* (MAUAM 5587); Jimma, Highlands north of the Gojeb River past Bersisi on the road to Jimma, ca. Delbey, 07º23'09" N, 036º51'49" E, 2210 m, on tree trunk, 11/11/2013, *B. Vigalondo et al.* *s.n.* (MAUAM 5590). **Amhara**, S. Gonder, Mountains between Bahir Dar and Gondar, about 5 km NW of Addis Zemen, 12º08'38'' N, 037º44'58'' E, 2225 m, on the trunk of a thorny shrub (Rhamnaceae), 15/11/2013, *F. Lara et al. 1311/47* (MAUAM 5591); N. Gonder, Simien Mts, Sankaber camp, 13º13'50" N, 038º02'27" E, 3250 m, on low branches of shrubby *Olea*, 17/11/2013, *V. Mazimpaka et al. s.n.* (MAUAM 5592); N. Gonder, Simien Mts, Gimbar river on the way from Ayna Medda camp to Guinch camp, 13º15'22" N, 038º06'42" E, 3316 m, on branches of a *Hypericum* tree, 18/11/2013*, B. Vigalondo et al.* *s.n.* (MAUAM 3634, 3635); *ibidem*, on trunk of *Erica*, 18/11/2013*, V. Mazimpaka et al. s.n.* (MAUAM 3673); N. Gonder, Simien Mts, way from Ayna Medda camp to Guinch camp, 13º15'30" N, 038º06'41" E, 3400 m, on bent trunk of *Lobelia rhynchopetalum*, *F. Lara et al.* *1311/68* (MAUAM 3674); N. Gonder, Simient Mts, upper part of the forest below Guinch camp, 13º15'50'' N, 038º06'34'' E, 3515 m, branches of *Erica arborea*, 18/11/2013, *B. Vigalondo et al. s.n.* (MAUAM 5588); N. Gonder, Simien Mts, around Chenek Camp, 13º15'38" N, 038º11'32" E, 3595 m, on branches of a *Hypericum* tree, 19/11/2013, *F. Lara et al.* *1311/80* (MAUAM 5594); N. Gonder, Simien Mts, Chenek Camp, south slope near the camp, 13º15'22" N, 038º11'29" E, 3570 m, on trunks of *Lobelia rhynchopetalum*, 19/11/2013, *B. Vigalondo et al*. *s.n.* (MAUAM 5595); N. Gonder, Simien Mts, hillside above Hollywater, 13º15'54" N, 038º12'13" E, 3900 m, on shaded *Erica arborea* crown, 19/11/2013, *B. Vigalondo et al.* *s.n.* (MAUAM 5596); N. Gonder, Simien Mts, Ambaras, Guimbar river, tributary side valley, 13º15'22" N, 038º06'45" E, 3340 m, on branches of *Erica* sp., 20/11/2013, *F. Lara et al.* *1311/92* (MAUAM 3671); N. Gonder, Simien Mts, Ambaras, Guimbar river, tributary side valley on the northern exposure slope, 13º15'18" N, 038º06'48" E, 3440 m, on trunk of a *Hypericum* tree, 20/11/2013, *B. Vigalondo et al.* *s.n.* (MAUAM 5597). **Southern Nations, Nationalities, and Peoples' Region (SNNPR)**, Dawro, Near Gena Bosa, 1 Km NW on the Waka-Issara road, 07º01'01" N, 037º14'05" E, 2330 m, on trunks of *Acacia* sp., 11/11/2013, *F. Lara et al.* *1311/39* (MAUAM 5589). **INDIA**. **Tamil Nadu**, Nilgiris Dist., Udhagamandalam (Ooty), Thalayathimund, New Dodabetta Rd, 11º24'14" N, 076º43'13" E, 2385 m, on a concrete pole with north orientation in urbanized area, 30/12/2018, *F. Lara 1812/43* (MAUAM 3639); Nilgiris Dist., Udhagamandalam (Ooty), Vannarapettai, Ooty Botanical Garden, 11º25'08" N, 076º42'39" E, 2230 m, on branches of *Ligustrum lucidum*, 31/12/2018, *F. Lara & J. Lara 1812/53* (MAUAM 3643); *ibidem*, on trunks of *Cordiline australis*, 31/12/2018, *F. Lara et al.* *1812/51* (MAUAM 3642); *ibidem*, on branches of *Acer negundo*, 31/12/2018, *F. Lara et al.* *1812/50* (MAUAM 3641); *ibidem*, on ornamental tree twigs, 31/12/2018*, F. Lara et al.* *1812/52* (MAUAM 3638); Dindigul Dist., Kodaikanal, Coaker's Walk, Kodai Resort Hotel, 10º13'59" N, 077º29'40" E, 2085 m, on branch of *Prunus* sp., 3/1/2019, *F. Lara et al.* *1901/06* (MAUAM 3644); Dindigul Dist., Kodaikanal, Kodaikanal Lake, Lake Rd, 10º13'50" N, 077º29'13" E, 2100 m, on tree trunk, 3/1/2019*, F. Lara et al.* *1901/11* (MAUAM 3646); Dindigul Dist., Kodaikanal, Kodaikanal Lake, Lake Rd, 10º14'00" N, 077º29'10" E, 2100 m, on trunk of *Alnus* sp., 3/1/2019, *F. Lara & J. Lara* *1901/12* (MAUAM 3647). **KENYA**. **Meru Co.**, Mt. Kenya,

**Annex 2.** Samples included in the GBS analyses and their corresponding barcodes. Information regarding the number of loci recovered and the missing data percentage is included.

| **Taxa** | **DNA code** | **Herbarium code** | **Library barcode** | **N Loci recovered** | **Missing data** |
| --- | --- | --- | --- | --- | --- |
| *L. firma* complex morphotype 1 | RDLF006 | **MAUAM 3632** | ATTG | 234 | 84.4 |
| *L. firma* complex morphotype 1 | RDLF011b | **MAUAM 3637** | TGCG | 373 | 91.0 |
| *L. firma* complex morphotype 1 | RDLF028 | **MAUAM 5553** | TTCTA | 324 | 90.1 |
| *L. firma* complex morphotype 1 | RDLF036 | **MAUAM 5571** | TAATG | 480 | 87.6 |
| *L. firma* complex morphotype 2 | RDLF009b | **MAUAM 3635** | CGGT | 1544 | 62.7 |
| *L. firma* complex morphotype 2 | RDLF023 | **MAUAM 5580** | CGGCA | 1459 | 60.4 |
| *L. firma* complex morphotype 2 | RDLF024 | **MAUAM 5588** | GAAGA | 1407 | 66.0 |
| *L. firma* complex morphotype 2 | RDLF027 | **MAUAM 5591** | ACGAG | 858 | 76.5 |
| *L. firma* complex morphotype 3 | RDLF013 | **MAUAM 3639** | GTAT | 1473 | 66.5 |
| *L. firma* complex morphotype 3 | RDLF014 | **MAUAM 3641** | AACCA | 934 | 80.3 |
| *L. firma* complex morphotype 3 | RDLF015 | **MAUAM 3642** | CCACG | 1200 | 65.4 |
| *L. firma* complex morphotype 3 | RDLF018 | **MAUAM 3646** | TATAA | 763 | 86.0 |
| *L. firma* complex morphotype 3 | RDLF019 | **MAUAM 3647** | GAGCG | 1583 | 63.1 |
| *L. firma* complex morphotype 4 | RDLF001 | **MAUAM 5613** | AACT | 1355 | 44.2 |
| *L. firma* complex morphotype 4 | RDLF021 | **MAUAM 3631** | CTCAG | 1310 | 60.2 |
| *L. firma* complex morphotype 4 | RDLF035 | **MAUAM 4591** | GCCAA | 428 | 89.5 |
| *L. firma* complex morphotype 4 | RDLF0067 | **MAUAM 5603** | TGGCAA | 1590 | 61.2 |
| *L. firma* complex morphotype 4 | RDLF037 | **MAUAM 5606** | GTTCA | 1416 | 69.6 |
| *L. firma* complex morphotype 4 | RDLF022 | **MAUAM 5614** | TCTGG | 1061 | 65.5 |
| *Lewinskya affinis* (Brid.) F. Lara, Garilleti & Goffinet | RDLF057 | **MAUAM 3619** | TGAACA | 352 | 88.7 |
| *Lewinskya arborescens* (Thér. & Naveau) F. Lara, Garilleti & Goffinet | RDLF059 | **MAUAM 1894** | ATCGCA | 937 | 69.1 |
| *Lewinskya armata* (Lewinsky & van Rooy) F. Lara, Garilleti & Goffinet | RDLF058c | **MAUAM 4649** | CTGCCA | 235 | 95.0 |
| *Lewinskya fastigiata* (Bruch ex Brid.) Vigalondo, F. Lara & Garilleti | RDLF063 | **MAUAM 4895** | GAATCA | 391 | 90.9 |
| *Lewinskya galiciae* (F. Lara, Garilleti & Mazimpaka) F. Lara, Garilleti & Goffinet | RDLF054b | **MAUAM 4592** | CATGCA | 492 | 89.3 |
| *Lewinskya galiciae* (F. Lara, Garilleti & Mazimpaka) F. Lara, Garilleti & Goffinet | RDLF032b | **MAUAM 5554** | AGTCG | 668 | 78.6 |
| *Lewinskya graphiomitria* (Müll. Hal. ex Beckett) F. Lara, Garilleti & Goffinet | RDLF042c | **MAUAM 4882** | ATAGG | 500 | 86.5 |
| *Lewinskya hookeri* (Wilson ex Mitt.) F. Lara, Garilleti & Goffinet | RDLF061b | **MAUAM 5620** | TAACCG | 637 | 88.5 |
| *Lewinskya incurvomarginata* (Lewinsky & van Rooy) F. Lara, Garilleti & Goffinet | RDLF052b | **MAUAM 2949** | GCTCCA | 265 | 95.4 |
| *Lewinskya leptocarpa* (Bruch & Schimp. Ex Müll.Hal.) Vigalondo, F.Lara & Garilleti | RDLF045b | **MAUAM 5060** | CGATT | 544 | 86.6 |
| *Lewinskya shawi*i (Wilson) F. Lara, Garilleti & Goffinet | RDLF049b | **MAUAM 1895** | TGCAT | 306 | 90.9 |
| *Lewinskya tanganyikae* (P. de la Varde) F. Lara, Garilleti & Goffinet | RDLF046c | **MAUAM 4593** | AATGT | 587 | 88.3 |
